# Celebrating the founding editor of *Brain Communications*: Tara Spires-Jones

**DOI:** 10.1093/braincomms/fcag139

**Published:** 2026-05-05

**Authors:** David Belin

**Affiliations:** Cambridge, UK

## Abstract

Our incoming editor-in-chief pays tribute to the founding editor of *Brain Communications*, Professor Tara Spires-Jones.

Welcome to Volume 8, Issue 3 of *Brain Communications*, which marks a milestone in the life of our journal, namely the transition in leadership from our founding editor, Tara Spires-Jones, to me as new editor-in-chief. Such transitions are important moments in the life of a journal, inviting both reflection on what has been accomplished and anticipation of what lies ahead. In the present case, it serves as a reminder that when you rest on the shoulders of giants, you lose sight of how big their shoes are and how difficult, if at all possible, they are to fill.

Seven years ago, Tara took on the challenge of founding a new charity-owned journal in translational neuroscience at a time when many society journals in neuroscience, such as *Neuroscience*, *Journal of Neuroscience* and *European Journal of Neuroscience*, the flagship outlets of the International Brain Research Organisation (IBRO), the Society of Neuroscience (SFN) and the Federation of European Societies of Neuroscience (FENS), respectively, were already suffering the consequences of the emergence of several for-profit entities that were siphoning away some of their content.

*Brain Communications* is the fully open access sister journal to *Brain*, which supports the charitable mission of Guarantors of Brain, one of the oldest neuroscience societies in the world. It was founded to respond to the transition from the historic, but relatively closed, model of subscription-dependent, paper-based, peer-reviewed journals to that of a dissemination of scientific knowledge to all, without a paywall. At the helm, Tara brought together a diverse group of highly motivated associate and advisory editors and a wider community, including *Brain Communications’* scientific editor, Manuela Marescotti, and a dedicated managing editor, Catriona Byres. This team, alongside the journal’s authors and reviewers, shaped one of the most respectful, supportive and scientifically innovative communities in translational neuroscience of the past decade. In doing so, Tara has played a transformative role in establishing a journal that reflects the evolving values of modern neuroscience: rigour, transparency, inclusivity and accessibility.

Tara has also nurtured the *Brain Communications’* editorial office team, which has grown, with the journal, since the early days to more than 10 members, now including PhD students, interns and editorial staff. As a manager, Tara is as professional and conscientious as she is in her other endeavours. She fosters a healthy work environment, respecting each role and supporting professional growth. Tara pays attention to individual aspirations and career plans. Her friendly yet resolute temperament has been the ‘glue’ for the international editorial board and editorial office of *Brain Communications*, not only ‘in health’, but also during the difficult months of the pandemic, which struck within a year of the journal’s launch.

Launching a new scientific journal is never a trivial undertaking. It requires vision, credibility and an unwavering commitment to scientific quality. From its inception, *Brain Communications* benefited from Tara’s clear editorial philosophy: to create a forum which welcomes robust and reproducible neuroscience, values methodological transparency, and promotes constructive peer review. Tara gave her time without restraint to our journal’s community as the founding editor, shaping its ethos, its manuscript management pipeline, and establishing its reputation, while also creating a supportive environment for her associate editors, including myself, to test ideas, such as the Reviewer Academy that *Brain Communications* launched in 2021. Under Tara’s guidance, the journal rapidly became more than just a peer-reviewed publication outlet with a strong commitment to sound science and fair and author-friendly editorial practices: it is a platform for a culture of openness and scientific dialogue. I am incredibly proud and grateful to have been part of this adventure from the beginning.

This editorial leadership mirrors a broader pattern that has defined Tara’s career: a passion for neuroscience, particularly the study of Alzheimer’s disease, and an unmatched commitment to serving the scientific community and society. Tara achieved all of the above while being an incredibly active president of the British Neuroscience Association (BNA) between 2023 and 2025. During her presidency, she championed initiatives to increase diversity and inclusion in neuroscience, to strengthen engagement with policymakers, and to support early-career scientists. Through the BNA, she helped amplify the collective voice of neuroscientists across the UK, ensuring that neuroscience research remains visible and valued within society and government.

Equally significant has been her work as a science communicator and advocate. At a time when public trust in science must be actively nurtured, Tara has consistently demonstrated the importance of engaging with audiences beyond academia. Whether speaking at public events, participating in policy discussions, or promoting accessible communication of neuroscience discoveries, she has emphasized that scientific knowledge carries a responsibility: to be shared, explained, and placed within a broader societal context. Her advocacy extends to issues central to the future of research itself—open science practices, responsible publishing, and the need for supportive academic cultures.

These incredible contributions build upon her scientific achievements as a leading investigator in neurodegenerative disease research, particularly in understanding synaptic pathology in Alzheimer's disease. Tara has published hundreds of papers, which have attracted 30 000 citations to date. She was elected a fellow of the Academy of Medical Sciences in her mid-forties, and she has received more prestigious awards and accolades than I can count. But you would never know, as Tara is incredibly humble and cares only about science and service.

The success of *Brain Communications* stands as a testament to her vision.

It is therefore with humility that I step into the role of editor-in-chief of *Brain Communications*, succeeding one of the scientists whom I admire the most and whom I am very proud to call my friend. On behalf of the editorial office team, the editorial board, the authors and the reviewers of *Brain Communications*, thank you, Tara!

The cover image for this issue comes from Sanchez-Migallon *et al.*^1^ and shows triple immunofluorescence in the human amygdala in Huntington’s disease revealing huntingtin deposits, astroglia and microglia, with autofluorescence of lipofuscin also visible.
